# Differences between brachial and aortic blood pressure in adolescence and their implications for diagnosis of hypertension

**DOI:** 10.1097/HJH.0000000000003743

**Published:** 2024-04-24

**Authors:** Alun D. Hughes, George Davey Smith, Laura D. Howe, Deborah Lawlor, Siana Jones, Chloe M. Park, Nish Chaturvedi

**Affiliations:** a MRC Unit for Lifelong Health & Ageing, Department of Population Science & Experimental Medicine, Institute of Cardiovascular Science, University College London, London; b MRC Integrative Epidemiology Unit, University of Bristol, Oakfield House, Oakfield Grove, Bristol, UK

**Keywords:** adolescence, aortic, blood pressure, hypertension

## Abstract

**Objectives:**

Blood pressure (BP) is the leading global cause of mortality, and its prevalence is increasing in children and adolescents. Aortic BP is lower than brachial BP in adults. We aimed to assess the extent of this difference and its impact on the diagnosis of hypertension among adolescents.

**Methods:**

We used data from 3850 participants from a UK cohort of births in the early 1990s in the Southwest of England, who attended their ∼17-year follow-up and had valid measures of brachial and aortic BP at that clinic [mean (SD) age 17.8 (0.4) years, 66% female individuals]. Data are presented as mean differences [95% prediction intervals] for both sexes.

**Results:**

Aortic systolic BP (SBP) was lower than brachial SBP [male, −22.3 (−31.2, −13.3) mmHg; female, −17.8 (−25.5, −10.0) mmHg]. Differences between aortic and brachial diastolic BP (DBP) were minimal. Based on brachial BP measurements, 101 male individuals (6%) and 22 female individuals (1%) were classified as hypertensive. In contrast, only nine male individuals (<1%) and 14 female individuals (<1%) met the criteria for hypertension based on aortic BP, and the predictive value of brachial BP for aortic hypertension was poor (positive-predictive value = 13.8%). Participants with aortic hypertension had a higher left ventricular mass index than those with brachial hypertension.

**Conclusion:**

Brachial BP substantially overestimates aortic BP in adolescents because of marked aortic-to-brachial pulse pressure amplification. The use of brachial BP measurement may result in an overdiagnosis of hypertension during screening in adolescence.

## INTRODUCTION

High blood pressure (BP) is the leading global cause of mortality [1]. Elevated BP is frequently evident in youth, with recent studies reporting that around 2–13% of young people may be hypertensive, depending on geographical region and definition used to classify hypertension [2,3]. Disturbingly, there is evidence that the prevalence of elevated BP in children is increasing [4], probably as a result of the global epidemic of obesity and physical inactivity [5]. Elevated BP in young people is important as there is evidence that BP tracks into adulthood [6], and has long-term implications for cardiovascular health and mortality [7,8]. Conversely, a diagnosis of hypertension can have adverse psychological consequences [9] and pharmacological treatment for hypertension has adverse effects. This is particularly relevant because the benefits and harms of antihypertensive medication have not been studied extensively in young people [2,10].

It is well recognized that SBP can differ substantially depending on the site of measurement. Typically brachial SBP is higher than aortic SBP because of pulse pressure amplification due to wave reflection [11]. In adults, pulse pressure amplification is highly variable [12], tends to decrease with age [13] and can account for isolated systolic hypertension (ISH) [14]. ISH in adults between 18 and 39 years is more common than systolic plus diastolic hypertension [15], although its clinical significance is debated in this age group [16,17]. Further, there is evidence that aortic as opposed to brachial BP is a more relevant prognostic indicator – being more strongly associated with cardiovascular events in adults [18,19], and with more target organ damage in both adults [20] and adolescents [21].

At present, there is limited evidence on the extent of the difference between aortic and brachial BP in adolescents, the prevalence of ISH in people below the age of 18 years, or its impact on the diagnosis of hypertension in youth, an issue highlighted by recent international guidelines [3]. We therefore, aimed to determine the difference in brachial and aortic BP in a large sample of adolescents drawn from an English birth cohort, the Avon Longitudinal Study of Parents and Children (ALSPAC); and the proportion of people with hypertension or ISH in this sample based on the use of brachial or aortic BP. A further aim was to determine whether aortic and brachial BP, hypertension, and ISH differed in relation to target organ damage to the heart [i.e. left ventricular mass index (LVMI)] [21,22] or vasculature (i.e. carotid intima–media thickness (cIMT) [23] and carotid–femoral pulse wave velocity (cfPWV)) [24], and to investigate possible factors that contributed to aortic to brachial pulse pressure amplification in adolescents.

## METHODS

### Study design and participants

Pregnant women resident in the former county of Avon, Southwest England, with expected dates of delivery from 1 April 1991 to 31 December 1992 were invited to participate in The Avon Longitudinal Study of Parents and Children (ALSPAC). The initial number of pregnancies enrolled was 14 541 (for these, at least one questionnaire was returned or a ‘Children in Focus’ clinic was attended by 19 July 1999). Of these initial pregnancies, there were 14 062 live births and 13 988 children who were alive at 1 year of age and have been followed since then [25,26]. The study website (http://www.bristol.ac.uk/alspac/researchers/our-data/) contains details of all available data. The present analysis was based on 5081 participants aged ∼17 years who attended the ALSPAC F17 clinic between 2009 and 2011 as part of an ongoing follow-up. Ethical approval was obtained from the ALSPAC Law and Ethics Committee and Local Research Ethics Committee, and all participants provided written informed consent.

Individuals with diabetes mellitus (*n* = 21), familial hypercholesterolemia (*n* = 8), known heart disease (*n* = 3), pregnancy (*n* = 15), or those who did not participate in the BP measurement session for any reason (*n* = 741) were excluded. A further 11 participants refused tonometry measurements, and in 392 participants, tonometry measurements failed quality control procedures, yielding a total of 3850 evaluable recordings (Supplementary Figure S1).

### Clinic measurements

Patient age and sex were recorded at the clinic. Demographic and lifestyle data were obtained using a questionnaire. Socioeconomic position was assessed based on the father's occupation (using the 1991 UK Office of Population Censuses and Surveys classification) and mother's education ((less than O-level, O-level, or more than O-level but no degree, and degree or above, where O-levels were the standard school-leaving qualifications taken around age 16 years until recently in the UK). Alcohol consumption was assessed as the number of drinks containing alcohol consumed on a typical drinking day, and smoking was categorized as never, ever but not currently, or currently. Weight and height were measured while the participants wore light clothing and no shoes. Height was estimated to the nearest 0.1 cm with a Harpenden Stadiometer. Body weight was measured to the nearest 0.1 kg using a Tanita TBF 305 scale. Body composition was assessed using a Lunar Prodigy Dual-energy X-ray absorptiometry scanner (GE Medical Systems, Madison, Wisconsin, USA). Habitual physical activity was assessed using a hip-worn uniaxial ActiGraph device between the age 14 and 17 years (AM7164 2.2; ActiGraph LLC, Fort Walton Beach, Florida, USA) and average daily minutes of total physical activity at light, moderate, or vigorous intensity was calculated based on cut-points of 200–3599, 3600–6199, and ≥6200 cpm, respectively. cfPWV was measured using a Vicorder device (SMT Medical Technology GmbH, Bristol, UK) as previously described [27]. cIMT was measured in left and right common carotid arteries by ultrasound using a linear 12 MHz transducer (Vivid7, GE Medical, Chicago, Illinois, USA) and averaged [28]. Left ventricular mass was measured in approximately one in two participants selected in a quasi-random fashion using an ultrasound device (HDI 5000, Philips Healthcare, North Andover, Massachusetts, USA) equipped with a P4-2 Phased Array ultrasound transducer according to the American Society of Echocardiography guidelines, as previously described and indexed to height^1.7^[29]. Blood samples were collected following an overnight fast for those assessed in the morning or a minimum of 6 h fasting for those assessed in the afternoon. Samples were centrifuged immediately, separated, and frozen at − 80 °C before analysis. Lipid profiles [total cholesterol, high-density lipoprotein (HDL) cholesterol, triglycerides], glucose, and insulin were measured as described previously [30]. Insulin sensitivity (HOMA-S) was estimated using the Homeostasis Assessment Model (Version 2.2.3) [31].

The participants’ sitting BP and heart rate were measured at least three times with at least a minute interval using an Omron 705IT device according to contemporary guidelines in the dominant arm using an appropriate cuff size [32]. The average of the final two readings was used. The BP waveform was measured using radial tonometry (SphygmoCor, AtCor Medical), and aortic pressure was estimated using a generalized transfer function (GTF), which has been validated in adults and children [12,33]. The late systolic shoulder (SBP2) was also used as an alternative estimate of aortic SBP, which does not rely on a GTF [34]. Amplification was calculated as brachial pulse pressure/aortic pulse pressure. All measurements were made by trained investigators and ongoing quality control was conducted throughout the study; reproducibility was excellent, as has been reported previously [35].

In accordance with European Society of Hypertension (ESH) guidelines [36], hypertension was defined as brachial BP at least 140/90 mmHg. ISH was defined as SBP at least 140 mmHg and DBP less than 90 mmHg [36]. For the classification of hypertension based on aortic BP, we used the definition of aortic BP at least 130/90 mmHg as hypertensive [37,38], and aortic SBP at least 130 mmHg and DBP less than 90 mmHg as indicative of aortic ISH (aISH). A subsidiary analysis using the recent American Academy of Pediatrics Clinical Practice Guideline recommendations [2] (stage I hypertension: ≥130/80 mmHg) was also performed.

### Statistical analysis

All analyses were performed in Stata/MP 17.0 (StataCorp). Descriptive statistics for continuous variables are presented as mean (SD), and *N* (%) for categorical variables. Comparisons between the included sample and those eligible but not included were made using Student's *t* tests or chi^2^ tests as appropriate. Analyses were stratified by sex, based on previous evidence of sex differences in the aortic BP waveform and amplification [39]. For the main analysis of difference in brachial and aortic BP we used unadjusted linear regression or two-dimension fractional polynomial if there was evidence of nonlinearity. If there was evidence of heteroscedasticity, standard errors for the linear model were estimated using the bootstrap estimator. Linear regression results were summarized as beta coefficients with 95% confidence intervals (95% CI). When nonlinearity and heteroskedasticity were present, we used a combination of fractional polynomials and quantile regression to produce median, 5%, and 95% quantile boundaries for the nonlinear relationship (95% QI). For differences in the proportions of different definitions of hazard ratio using aortic and brachial BP, we used unadjusted logistic regression. We estimated the sensitivity, specificity, and positive and negative prediction metrics for hypertension and ISH by using aortic and brachial BP. Associations between aortic or brachial hypertension in the absence of aortic hypertension and measures of target organ damage were adjusted for potential confounders (age, BMI, fat and lean mass, habitual physical activity, maternal education, and socioeconomic status) chosen on the basis of background knowledge. To investigate potential predictors of amplification, we used a linear model with inclusion of clinical predictors (see Table 1), and model selection was performed using an elastic net with 10-folds. We report the percent variation explained in the outcome for all selected variables.

**TABLE 1:** Participant characteristics

	All			Male			Female		
Variable	*N*	Mean/%	SD	*N*	Mean/%	SD	*N*	Mean/%	SD
Age (years)	3850	17.8	0.4	1704	17.8	0.4	2146	17.8	0.4
Height (cm)	3767	171.3	9.4	1672	178.9	6.7	2095	165.2	6.2
Weight (kg)	3771	67.0	13.5	1675	72.3	12.9	2096	62.9	12.4
BMI (kg/m^2^)	3767	22.8	4.0	1672	22.6	3.7	2095	23.0	4.2
Fat mass (kg)	3706	18.2	10.4	1650	13.9	9.7	2056	21.8	9.6
Lean mass (kg)	3706	45.7	10.0	1650	55. 3	6.1	2056	38.0	4.3
Fasting glucose (mmol/l)	2562	5.0	0.4	1233	5.1	0.41	1329	4.9	0.4
Insulin (pmol/l)	2528	49.4	42.2	1223	45.5	36.2	1305	53.0	46.9
HOMA-S (%)	2513	147.2	81.6	1212	160.5	89.1	1301	134.9	71.9
Total cholesterol (mmol/l)	2562	3.8	0.7	1233	3.6	0.6	1329	3.9	0.7
HDL-cholesterol (mmol/l)	2562	1.3	0.3	1,233	1.2	0.3	1329	1.3	0.3
Triglycerides (mmol/l)	2562	0.8	0.4	1233	0.8	0.4	1329	0.8	0.3
MVPA (min/day)	1488	23.5	18.6	632	30.3	20.4	856	18.6	15.3
Brachial SBP (mmHg)	3850	116.7	11.5	1704	122.6	10.7	2146	111.9	9.8
Brachial DBP (mmHg)	3850	64.6	7.5	1704	64.3	7.6	2146	64.8	7.5
Heart rate (bpm)	3850	69.7	10.8	1704	66.6	10.6	2146	17.8	0.4
Carotid–femoral PWV (m/s)	3107	5.8	0.7	1398	6.0	0.7	1709	5.5	0.6
Carotid–radial PWV (m/s)	3118	7.9	1.2	1399	8.0	1.3	1719	7.8	1.1
Room temperature (°C)	3286	21.6	2.0	1473	21.7	1.9	1813	21.6	2.0
Male sex	1704	44.3%							
Father's occupation	3465			1551			1914		
I – professional	386	11.1%		175	11.3%		211	11.0%	
II – managerial and technical	1340	38.7%		605	39.0%		735	38.4%	
IIINM – skilled nonmanual	410	11.8%		196	12.6%		214	11.2%	
IIIM – skilled manual	986	28.5%		408	26.3%		578	30.2%	
IV – partly skilled	249	7.2%		130	8.4%		119	6.2%	
V – unskilled	94	2.7%		37	2.4%		57	3.0%	
Mother's education	3496			1551			1945		
Less than O-level	671	19.2%		278	17.9%		393	20.2%	
O-level	1177	33.7%		502	32.4%		675	34.7%	
More than O-level but no degree	986	28.2%		456	29.4%		530	27.3%	
Degree or above	662	18.9%		315	20.3%		347	17.8%	
Smoking	3,279			1450			1829		
Never	1636	49.9%		781	53.9%		855	46.8%	
Ex	742	22.6%		304	21.0%		438	24.0%	
Current	901	27.5%		365	25.2%		536	29.3%	
Alcohol, drinks per typical drinking day	3191			1403			1788		
0	164	5.1%		5.2	5.2%		91	5.1%	
1 or 2	655	20.5%		298	21.2%		357	20.0%	
3 or 4	862	27.0%		368	26.2%		494	27.6%	
5 or 6	794	24.9%		310	22.1%		484	27.1%	
7 to 9	454	14.2%		226	16.1%		228	12.8%	
10 or more	262	8.2%		128	9.1%		134	7.5%	

BP, blood pressure; HOMA-S, homeostasis model assessment of insulin sensitivity; MVPA, time spent in moderate or vigorous physical activity; PWV, pulse wave velocity.

Primary analyses were performed using listwise deletion to handle missing data, on the assumption that conditional independence between missingness and aortic-to-brachial pulse pressure amplification (outcome) was more plausible than a missing-at-random assumption.

## RESULTS

The participants’ characteristics are listed in Table 1. Sixty-six percent of the participants were female participants, and the mean age for both sexes was 17.8 years. The mean brachial BP for both sexes was 116.7/64.6 mmHg, with male individuals having a higher brachial BP than female individuals. Compared with all those invited, participants who attended clinics were more likely to be female individuals and come from more advantaged socioeconomic circumstances (Supplementary Table S1).

**FIGURE 1:**
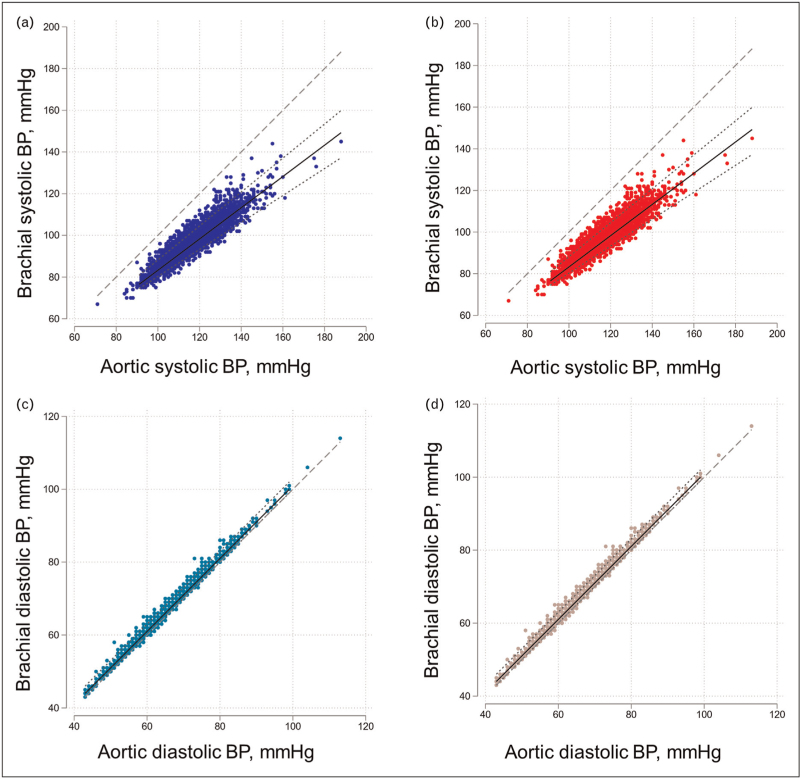
Relationship between brachial and aortic BP. (a) SBP (male), (b) SBP (female), (c) DBP (male), (d) DBP (female). Lines are fractional polynomial fits (solid line) to the data with 5 and 95% quantile limits (dotted line). The dashed line is the line of unity.

Figs. 1a–d show a comparison of brachial and aortic SBP and DBP in male and female individuals. The distribution of differences in brachial and aortic SBP between male and female individuals is shown in Supplementary Figure S2.

For both male and female individuals, there was a difference between aortic and brachial SBP (male individuals: −22.3 [−31.2, −13.3] mmHg; female individuals: − 17.8 [−25.5, −10.0] mmHg). The difference between aortic and brachial SBP was attributable to large aortic to brachial amplification which was of similar magnitude in both sexes [male: median 1.71 (range 1.23–1.96); female: median 1.71 (range 1.10–2.08)], although because BP in male individuals was higher absolute differences were larger for male than female individuals. There was no evidence of a difference in the slope of the relationship between the aortic and brachial SBP (*P* = 0.13). Differences between SBP2, an alternative measure of aortic SBP and brachial SBP, were even larger (male: −33.7 [−53.0, −14.4] mmHg; female: −25.0 [−40.7, −9.2] mmHg), with larger differences between males and females.

In contrast, there was a minimal difference between aortic and brachial diastolic BP in both sexes (male: 1.4 [−0.4, 3.1] mmHg; female: 1.2 [−0.3, 2.8] mmHg) (Fig. 1c and d).

### Classification and prevalence of hypertension using brachial and aortic blood pressure

On the basis of brachial BP, 123 (3%) participants were classified as hypertensive; of these ISH accounted for 109 (88%). There was a marked sex difference in hypertension prevalence based on brachial BP, with 101 hypertensive male individuals (6% of males) and 22 hypertensive female individuals (1% of females). ISH accounted for 98 (97%) cases of hypertension in male individuals and only 11 (50%) cases of hypertension in female individuals.

When aortic BP was used, only 23 patients (0.6%) had aortic hypertension. Of these, five (21%) were due to aISH. A minimal sex difference was observed for aortic hypertension: nine male individuals (0.5%) had aortic hypertension, of which four were attributable to aISH. In contrast, 14 female individuals (0.7%) had aortic hypertension, of which only one had aISH.

The classification matrices for aortic and brachial hypertension are presented in Table 2. Although the sensitivity, specificity, and negative-predictive value of brachial BP for aortic hypertension were excellent, brachial BP had a poor positive-predictive value for aortic hypertension (13.8%).

**TABLE 2:** Classification matrix for aortic and brachial SBP according to the ESH guideline classification

	Brachial BP		
Aortic BP	Hypertensive	Normotensive	Total
Aortic hypertensive	17	2	19
Aortic normotensive	106	3725	3831
Total	123	3727	3850

Measure	Estimate	95% confidence interval
Sensitivity	89.5%	66.9–98.7%
Specificity	97.2%	96.7–97.7%
Likelihood ratio (+)	32.3	25.0–41.0
Likelihood ratio (−)	0.11	0.03–0.40
Positive-predictive value	13.8%	8.3–21.2%
Negative-predictive value	99.9%	99.8–100%

The results for ISH were similar (Table 3) with excellent sensitivity, specificity, and negative-predictive value of brachial BP for aortic ISH but very poor positive-predictive value for aortic ISH (4.6%).

**TABLE 3:** Classification matrix for aortic and brachial isolated systolic hypertension

	Brachial BP		
Aortic BP	ISH	No ISH	Total
Aortic ISH	5	0	5
Aortic no ISH	104	3741	3845
Total	109	3741	3850

Measure	Estimate	95% confidence interval
Sensitivity	100%	47.8–100%
Specificity	97.3%	96.7–97.8%
Likelihood ratio (+)	37.0	30.6–44.7
Likelihood ratio (−)	0	–
Positive-predictive value	4.6%	1.5–10.4%
Negative-predictive value	100%	99.9–100%

BP, blood pressure; ISH, isolated systolic hypertension.

The results using a threshold of 130/80 mmHg for diagnosis of hypertension as recommended by the American Academy of Pediatrics Clinical Practice Guideline are shown in the Supplementary Table S2; these showed excellent sensitivity and negative-predictive values but lower specificity (85.6%) and a poorer positive predictive value (4%) than the ESH-based criteria.

### Relationship of aortic hypertension compared with isolated systolic hypertension on early target organ damage in adolescence

Both aortic and brachial hypertension without aortic hypertension were associated with cardiac target organ damage (higher LVMI), although the elevation in LVMI was larger in patients with aortic hypertension than in those with ISH [13.4 (95% CI 4.0–22.8) g/m^1.7^; *P* = 0.005 versus 7.1 (95% CI 1.4–12.8) g/m^1.7^; *P* = 0.015] after adjustment for potential confounders. There was no convincing evidence that either brachial or aortic hypertension or ISH was associated with vascular target organ damage, as assessed by cIMT or cfPWV.

### Predictors of aortic to brachial pulse pressure amplification

For male individuals, four variables were identified as predictors of aortic to brachial pulse pressure amplification (age, heart rate, crPWV, and room temperature). The out-of-sample *r*^2^ was small (*r*^2^ = 0.04), indicating that it was largely unexplained by these variables. A sensitivity analysis excluding physical activity identified a total of eight predictors (age, total fat mass, HDL-cholesterol, heart rate, smoking, crPWV, cfPWV, and room temperature) that were only minimally different in terms of model fit (*r*^2^ = 0.08) despite the larger number of predictors and fewer missing values.

For female individuals, five variables (HDL-cholesterol, triglycerides, heart rate, maternal education, and cfPWV) were predictors of amplification; however, the out-of-sample *r*^2^ was very modest (*r*^2^ = 0.03). Sensitivity analysis excluding physical activity only marginally improved the variance explained by the model (*r*^2^ = 0.08).

## DISCUSSION

Brachial SBP was substantially higher than aortic SBP in both sexes in a birth cohort of adolescents and that this difference varied considerably between individuals. This difference in adolescents (male individuals: 22 mmHg, female individuals: 18 mmHg) was considerably higher than has been typically found in adults (∼12 mmHg [13,40]). Higher brachial SBP was attributable to pulse pressure amplification and led to a large discrepancy in hypertension diagnosis when criteria based on brachial and aortic BP were compared.

Both aortic hypertension and brachial hypertension without aortic hypertension, which corresponded to ISH in almost all instances, were associated with increased LVMI, although the association was stronger with aortic hypertension. There was no evidence of vascular target organ damage (based on PWV or cIMT) in people with hypertension or ISH. Penalized regression (elastic net) using a wide range of measured variables, (including age, heart rate, cfPWV, crPWV, and cardiovascular risk factors) explained less than 10% of the variance in amplification in either sex, indicating that differences between brachial SBP and aortic SBP cannot be predicted, at least by these variables; therefore, amplification is an important and unexplained source of variation in brachial SBP.

Our finding of large differences in brachial and aortic SBP because of amplification in young people is consistent with previous studies. In the Anglo-Cardiff Collaborative Trial II, younger age was by far the strongest predictor of amplification, with smaller contributions from sex, diabetes, smoking, and cardiovascular diseases [13]. As the age range in our birth cohort was narrow and participants were younger and predominantly healthy, our findings are broadly consistent with these observations.

Aortic to brachial pulse pressure amplification is attributable to wave reflection from the downstream circulation, which arises because of impedance mismatching in the peripheral arterial tree [11]. At an individual bifurcation, the magnitude of wave reflection depends on the geometrical and biophysical properties of the parent and daughter arteries [41], but reflection patterns from anatomically asymmetric trees such as those in the upper limb are complex [42] which probably explains why the magnitude of pulse pressure amplification is unpredictable between individuals.

The high variability in amplification between individuals in this age group results in discrepancies in the identification of hypertension based on aortic or brachial BP. These findings contribute to the debate regarding spurious hypertension in young people [16,17]. Although the definition of hypertension is arbitrary [43], and it is relatively uncommon in children and adolescents (current global estimates are around 2–13% [2,3]), its prevalence has risen sharply since the 1990s and there are calls for more screening [44]. Previous studies have reported that aortic BP is a better predictor of outcomes than brachial BP in adults, and we have reported previously in ALSPAC that aortic pulse pressure is more strongly associated with left ventricular hypertrophy than brachial pulse pressure in adolescence [21]. Another more recent study using ambulatory aortic BP in selected normotensive and hypertensive young people drew similar conclusions with regard to the superiority of aortic BP over brachial BP as a predictor of LV hypertrophy [45]. Although the evidence cannot be definitive in the absence of hard endpoints (which will take years to accrue), our data suggest that aortic BP may be a more appropriate indicator of risk in young people – this is consistent with most key organs being exposed to aortic, not brachial SBP. As cuff-based devices are now available for measurement of aortic pressure, their wider use in children and adolescents may support therapeutic decision-making.

Our findings of large variability in amplification may also help explain previous conflicting observations in young adults comparing the relationships of SBP and DBP with later cardiovascular mortality [46–48], as this variability is likely to introduce uncertainty in estimated risk relationships. Although aortic BP was a stronger predictor of early cardiac target organ damage than brachial BP, there was a weak association between ISH and higher LVMI in male individuals. This is consistent with previous work showing associations between systolic hypertension and increased LVMI in children [49].

Our study has several limitations. At recruitment, ALSPAC was not representative of the UK population [25], and it may also differ from more contemporary cohorts. Men were under-represented in the sample and, as with all cohort studies, there has been attrition over time, which may introduce further bias and limit the generalizability of the findings. Further studies in different geographic regions covering different age ranges during childhood would be valuable. We cannot exclude residual or unmeasured confounding in our analysis and the lack of measures of sedentary behaviours may be a particular limitation. In ALSPAC measurement of BP was made on only one occasion whereas a diagnosis of hypertension usually requires BP measurements on multiple occasions. This may have led to a higher proportion of people identified as having BP in the hypertensive range. Nevertheless, it should not have influenced the difference between brachial and aortic pressure [50], and the white coat effect is similar on brachial and aortic pressure, at least in adults [51]. The strengths of the study include its large sample size and the standardized measurements of BP and other cardiovascular indices.

In conclusion, BP measured at the brachial artery is higher than aortic BP in adolescents owing to marked aortic-to-brachial pulse pressure amplification. The use of brachial BP could result in an overdiagnosis of hypertension during adolescence.

## ACKNOWLEDGEMENTS

We are extremely grateful to all the families who took part in the study, the midwives for their help in recruiting them, and the ALSPAC team, which included interviews, computer and laboratory technicians, clerical workers, research scientists, volunteers, managers, receptionists, and nurses.

Funding: cardiovascular measurements at age 17 years were supported by grants from the Wellcome Trust (086676/7/08/Z) and the British Heart Foundation (PG/06/145 and CS/15/6/31468 and SP/F/21/150020). G.D.S., L.D.H., and D.L. worked within the MRC Integrative Epidemiology Unit at the University of Bristol, which is supported by the Medical Research Council (MC_UU_00011/1&6). The UK Medical Research Council and Wellcome Trust (102215/2/13/2) together with the University of Bristol provided core support for the ALSPAC study. The other authors declare no relevant financial relationships.

Role of the funder/sponsor: the funders had no role in the design or conduct of the study.

### Conflicts of interest

G.D.S. reports Scientific Advisory Board Membership for Relation Therapeutics and Insitro. N.C. receives remuneration for membership of data monitoring and safety committees for trials sponsored by AstraZeneca pharmaceuticals.

## Supplementary Material

Supplemental Digital Content
